# Alpha-Soluble NSF Attachment Protein Prevents the Cleavage of the SARS-CoV-2 Spike Protein by Functioning as an Interferon-Upregulated Furin Inhibitor

**DOI:** 10.1128/mbio.02443-21

**Published:** 2022-01-11

**Authors:** Jinliang Wang, Jie Luo, Zhiyuan Wen, Xinxin Wang, Lei Shuai, Gongxun Zhong, Chong Wang, Ziruo Sun, Weiye Chen, Jinying Ge, Renqiang Liu, Xijun Wang, Zhigao Bu

**Affiliations:** a State Key Laboratory of Veterinary Biotechnology, Harbin Veterinary Research Institute, Chinese Academy of Agricultural Sciences, Harbin, People’s Republic of China; Wuhan Institute of Virology, Chinese Academy of Sciences; The Peter Doherty Institute for Infection and Immunity

**Keywords:** SARS-CoV-2 spike protein, cleavage, alpha-soluble NSF attachment protein, furin inhibitor, interferon upregulated

## Abstract

Loss of the furin cleavage motif in the SARS-CoV-2 spike protein reduces the virulence and transmission of SARS-CoV-2, suggesting that furin is an attractive antiviral drug target. However, lack of understanding of the regulation of furin activity has largely limited the development of furin-based therapeutic strategies. Here, we find that alpha-soluble NSF attachment protein (α-SNAP), an indispensable component of vesicle trafficking machinery, inhibits the cleavage of SARS-CoV-2 spike protein and other furin-dependent virus glycoproteins. SARS-CoV-2 infection increases the expression of α-SNAP, and overexpression of α-SNAP reduces SARS-CoV-2 infection in cells. We further reveal that α-SNAP is an interferon-upregulated furin inhibitor that inhibits furin function by interacting with its P domain. Our study demonstrates that α-SNAP, in addition to its role in vesicle trafficking, plays an important role in the host defense against furin-dependent virus infection and therefore could be a target for the development of therapeutic options for COVID-19.

## INTRODUCTION

According to the World Health Organization (WHO), the COVID-19 pandemic has caused 177 million confirmed human cases and approximately 4 million deaths worldwide as of 19 June 2021. A novel coronavirus, named severe acute respiratory syndrome coronavirus 2 (SARS-CoV-2), which belongs to the betacoronavirus genus, was identified as the causative pathogen of the disease (1). The envelope Spike (S) protein of coronaviruses plays key roles in cell receptor binding and entry, transmissibility, and pathogenicity (2). SARS-CoV-2 shares similar genomic homology with SARS-CoV, and both viruses use human angiotensin-converting enzyme 2 (ACE2) as a binding receptor (3–5). SARS-CoV-2 S protein is synthesized as a precursor and must be cleaved into its S1 and S2 subunits by furin to perform its function (6). The furin cleavage motif (-RRAR-) of the S protein of SARS-CoV-2 is similar to that of Middle Eastern respiratory syndrome-related coronavirus (MERS-CoV) but differs from that of SARS-CoV (7, 8).

The furin cleavage motif has been shown to be important for the biological properties of SARS-CoV-2. Deletion of the furin cleavage motif in the S protein decreases SARS-CoV-2 replication in human respiratory cells and reduces the virulence and transmissibility of SARS-CoV-2 in animal models (9, 10). Structural analysis has shown that furin cleavage allows a higher proportion of SARS-CoV-2 S protein to bind to the human ACE2 receptor (11), and a study by Cheng et al. revealed that the D614G substitution in the S protein increases SARS-CoV-2 transmission by enhancing the cleavage efficacy of the S protein (12). Exploring how cells regulate furin function during SARS-CoV-2 infection will provide important information about SARS-CoV-2 pathogenesis and help in the development of therapeutic strategies for COVID-19. Here, we found that the cellular protein alpha-soluble NSF attachment protein (α-SNAP) is an important regulator of furin activity and is upregulated in the SARS-CoV-2 infected cells, where it exerts antiviral effects by inhibiting the cleavage of the SARS-CoV-2 S protein and restricting SARS-CoV-2 infection.

## RESULTS

### α-SNAP inhibits the cleavage of the SARS-CoV-2 S protein.

**FIG 1:**
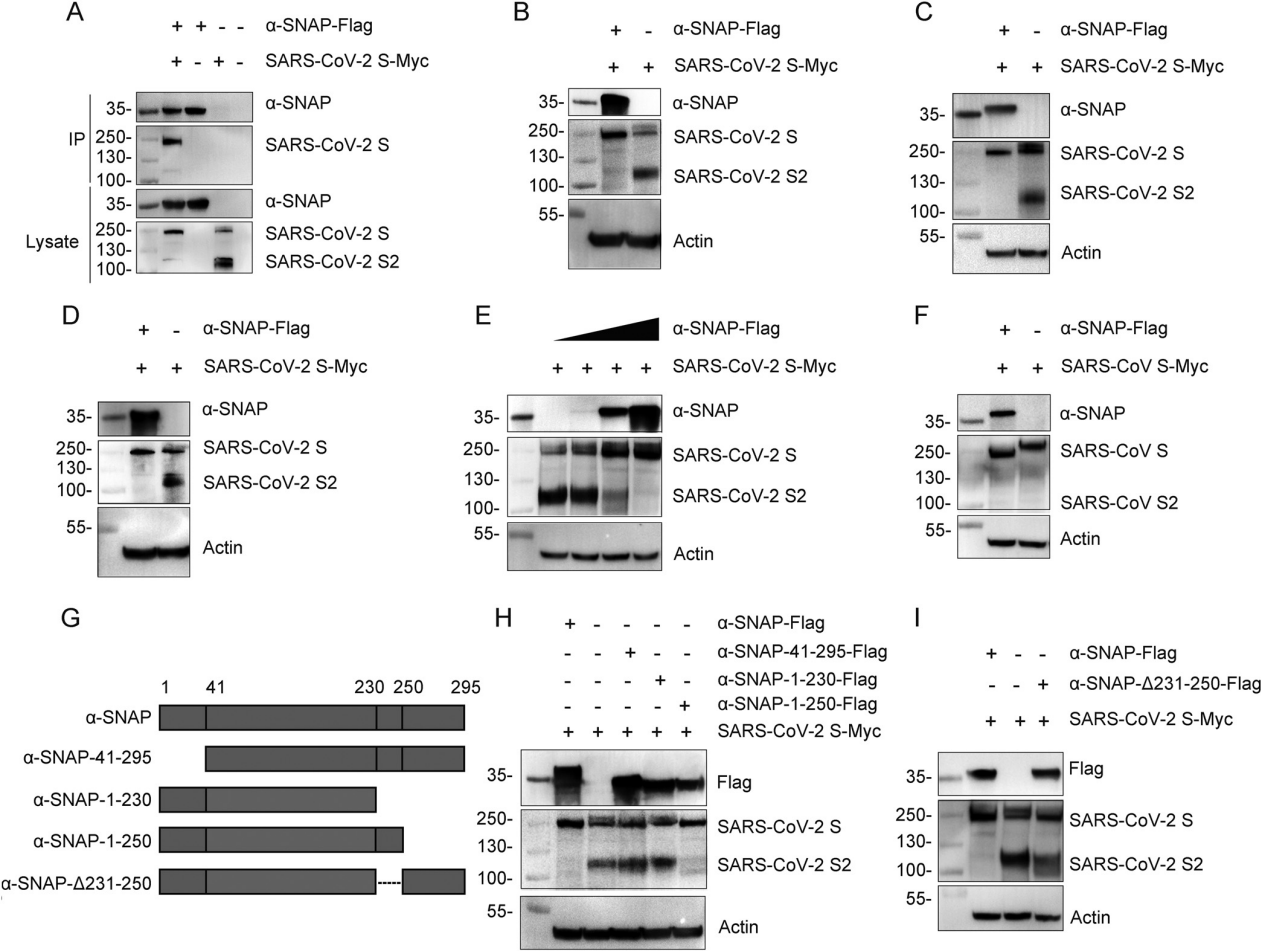
Overexpression of α-SNAP inhibits cleavage of the SARS-CoV-2 S protein. (A) α-SNAP-Flag and SARS-CoV-2 S-Myc were cotransfected into HEK293 cells and then immunoprecipitated by using anti-Flag agarose beads. (B to D) α-SNAP-Flag and SARS-CoV-2 S-Myc were cotransfected into HEK293 cells (B), Vero-E6 cells (C), or A549 cells (D), and then the S protein and S2 subdomain were detected by immunoblotting for Myc tag. (E) Different amounts of α-SNAP-Flag (0.02, 0.2, and 2 μg) were cotransfected with SARS-CoV-2 S-Myc (2 μg) into HEK293 cells. Then the S protein and S2 subdomain were detected by immunoblotting for Myc tag. (F) α-SNAP-Flag and SARS-CoV S-Myc were cotransfected into HEK293 cells, and then the S protein and S2 subdomain were detected by immunoblotting for Myc tag. (G) Schematic of the deletion mutants of α-SNAP. (H and I) SARS-CoV-2 S-Myc was cotransfected with α-SNAP-Flag, α-SNAP-41-295-Flag, α-SNAP-1-230-Flag, or α-SNAP-1-250-Flag (H) or α-SNAP-Δ231-250-Flag (I) into HEK293 cells, and then the S protein and S2 subdomain were detected by immunoblotting for Myc tag.

The novel cellular protein α-SNAP was found to interact with the S protein of SARS-CoV-2 in our mass spectrometry analysis by using the S1 subdomain of S protein as bait. This interaction with the SARS-CoV-2 S protein was confirmed in coimmunoprecipitation assays (Fig. 1A). Surprisingly, we found that, compared to HEK293 cells transfected with SARS-CoV-2 S-Myc alone, the levels of the S2 subdomain were significantly reduced in HEK293 cells cotransfected with α-SNAP-Flag and SARS-CoV-2 S-Myc (Fig. 1A), suggesting that α-SNAP may affect the cleavage of the S protein of SARS-CoV-2.

To confirm this observation, we evaluated the protein levels in SARS-CoV-2 S-Myc- and α-SNAP-Flag-cotransfected HEK293 cells, Vero-E6 cells, and A549 cells by Western blotting and found that the levels of the S2 subdomain were significantly reduced in cells cotransfected with α-SNAP-Flag and SARS-CoV-2 S-Myc compared to those in cells transfected with SARS-CoV-2 S-Myc alone (Fig. 1B to D). Moreover, when HEK293 cells were transfected with SARS-CoV-2 S-Myc and different amounts of α-SNAP-Flag, the level of the S2 subdomain progressively decreased as the amount of α-SNAP-Flag was increased (Fig. 1E). These results indicate that α-SNAP does indeed inhibit the cleavage of the SARS-CoV-2 S protein. Notably, cotransfection of α-SNAP had no effect on the cleavage of the SARS-CoV S protein (Fig. 1F).

In mammals, α-SNAP has two isoforms: beta-soluble NSF attachment protein (β-SNAP), and gamma-soluble NSF attachment protein (γ-SNAP) (13). β-SNAP has high sequence similarity (>80%) with α-SNAP and is mainly expressed in brain (14). γ-SNAP is only about 25% identical in its amino acid sequence to α-SNAP and is ubiquitously expressed (13). We found that the overexpression of β-SNAP inhibits the cleavage of SARS-CoV-2 S protein; however, overexpression of γ-SNAP had no effect (see Fig. S1 in the supplemental material).

10.1128/mBio.02443-21.1 FIG S1 Overexpression of β-SNAP but not γ-SNAP inhibits cleavage of the SARS-CoV-2 S protein. SARS-CoV-2 S-Myc was cotransfected with α-SNAP-Flag, β-SNAP-Flag, or γ-SNAP-Flag into HEK293 cells, and then the S protein and S2 subdomain were detected by immunoblotting for Myc tag. Download FIG S1, TIF file, 0.6 MB. Copyright © 2022 Wang et al. 2022 Wang et al. https://creativecommons.org/licenses/by/4.0/ This content is distributed under the terms of the Creative Commons Attribution 4.0 International license.

To identify the domain of α-SNAP that inhibits the cleavage of the SARS-CoV-2 S protein, we generated a series of truncated α-SNAP constructs and fused them to a Flag tag (Fig. 1G). HEK293 cells were transfected with SARS-CoV-2 S-Myc and the different truncated α-SNAP constructs, and immunoblotting for SARS-CoV-2 S-Myc showed that the truncated α-SNAP bearing the N-terminal 40-residue deletion (α-SNAP-41-295-Flag) and the truncated α-SNAP bearing a residue 231 to 250 deletion (α-SNAP-Δ231-250-Flag) lost the ability to inhibit the cleavage of the SARS-CoV-2 S protein (Fig. 1H and I), indicating that both the N-terminal 40 residues and residues 231 to 250 of α-SNAP are required to inhibit the cleavage of the SARS-CoV-2 S protein.

### α-SNAP inhibits the cleavage of the SARS-CoV-2 S protein in an NSF-independent manner.

α-SNAP is a well-known component of the vesicle trafficking machinery. It couples the energy produced by *N*-ethylmaleimide-sensitive factor (NSF)-mediated ATP hydrolysis to induce conformational changes in soluble NSF attachment protein receptors (SNAREs) and leads to the disassembly of the NSF/α-SNAP/SNARE complex, which is the final step of all intracellular membrane trafficking events (15). The interaction between α-SNAP and NSF is essential for the final step of intracellular membrane trafficking. A previous study demonstrated that the C-terminal 45 residues of α-SNAP are crucial for its interaction with NSF (16). However, we found that the C-terminal 45 residues of α-SNAP are dispensable for inhibiting the cleavage of the SARS-CoV-2 S protein, suggesting that α-SNAP inhibits the cleavage of the SARS-CoV-2 S protein by a novel, vesicle trafficking-independent manner. To confirm this theory, we investigated whether NSF inhibits the cleavage of the SARS-CoV-2 S protein. HEK293 cells were transfected with SARS-CoV-2 S-Myc and NSF-Flag. Immunoblotting analysis showed that cotransfection of NSF did not affect the cleavage of the SARS-CoV-2 S protein (see Fig. S2). These results demonstrate that inhibiting the cleavage of the SARS-CoV-2 S protein is a newly identified function of α-SNAP that is independent of its canonical function in vesicle trafficking.

10.1128/mBio.02443-21.2 FIG S2 Overexpression of NSF does not inhibit cleavage of the SARS-CoV-2 S protein. SARS-CoV-2 S-Myc was cotransfected with α-SNAP-Flag or NSF-Flag into HEK293 cells, and then the S protein and S2 subdomain were detected by immunoblotting for Myc tag. Download FIG S2, TIF file, 0.6 MB. Copyright © 2022 Wang et al. 2022 Wang et al. https://creativecommons.org/licenses/by/4.0/ This content is distributed under the terms of the Creative Commons Attribution 4.0 International license.

### α-SNAP directly interacts with furin.

**FIG 2:**
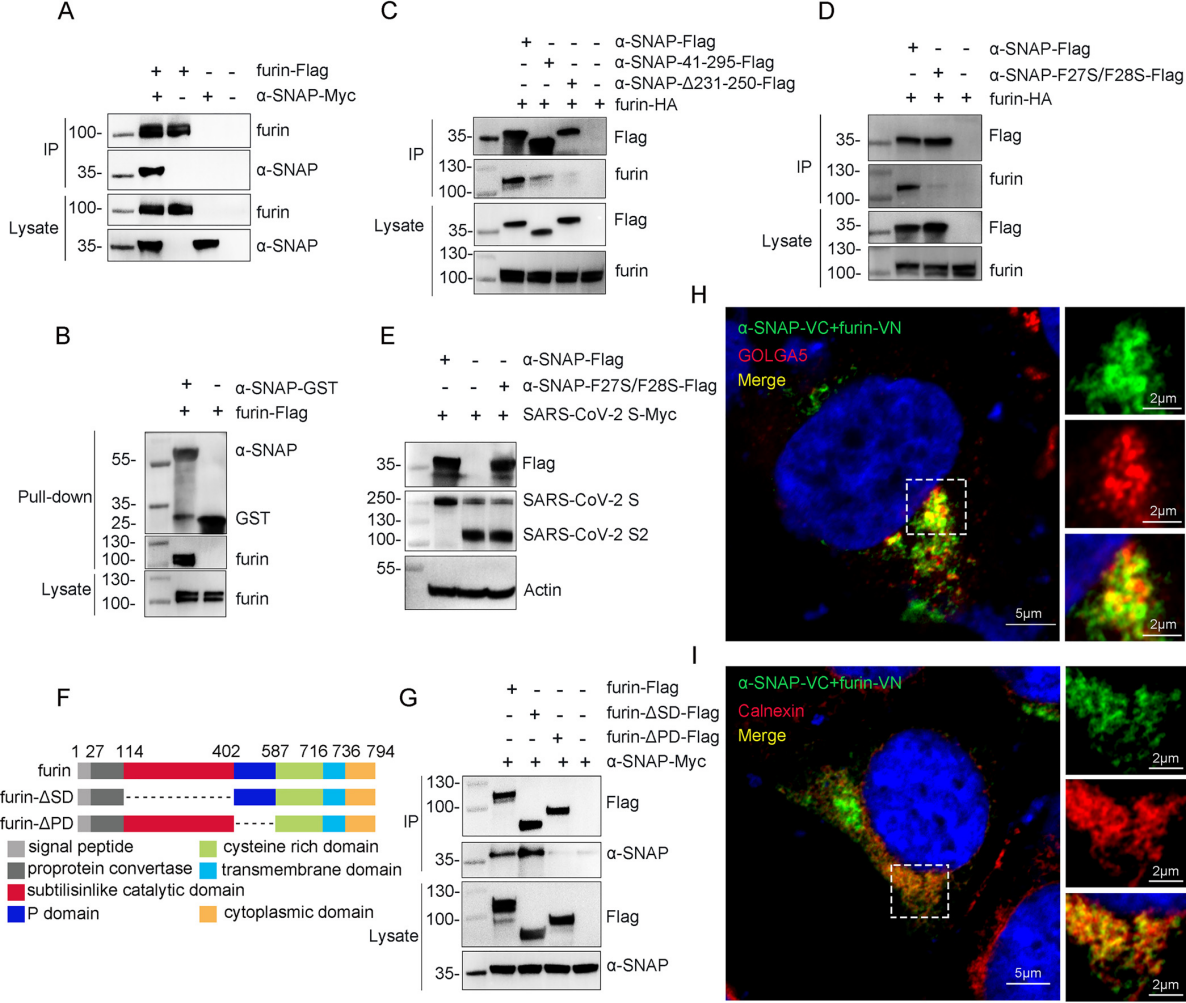
α-SNAP inhibits cleavage of the SARS-CoV-2 S protein by interacting directly with furin. (A) Furin-Flag coimmunoprecipitated with α-SNAP-Myc using anti-Flag agarose beads. (B) Purified α-SNAP-GST or GST was pooled with the lysate from furin-Flag-transfected HEK293 cells and then subjected to pulldown assays. (C and D) α-SNAP-Flag, α-SNAP-41-295-Flag, and α-SNAP-Δ231-250-Flag (C) or α-SNAP-F27S/F28S-Flag (D) coimmunoprecipitated with furin-HA by using anti-Flag agarose beads. (E) SARS-CoV-2 S-Myc was cotransfected with α-SNAP-Flag and α-SNAP-F27S/F28S-Flag into HEK293 cells, respectively, and then the S protein and S2 subdomain were detected by immunoblotting for Myc tag. (F) Schematic of the deletion mutants of furin. (G) Furin-Flag, furin-ΔSD-Flag, or furin-ΔPD-Flag coimmunoprecipitated with α-SNAP-Myc using anti-Flag agarose beads. (H and I) α-SNAP-VC and furin-VN were cotransfected into HEK293 cells, and then GOLGA5 (H) or calnexin (I) were stained by specific antibodies and analyzed by using confocal fluorescence microscopy.

The prototype proprotein convertase furin is responsible for the cleavage of the SARS-CoV-2 S protein (17). It is therefore reasonable to speculate that α-SNAP may interact with furin directly or indirectly and thereby regulate furin activity. To test this concept, HEK293 cells were cotransfected with furin-Flag and α-SNAP-Myc. Coimmunoprecipitation assays showed that α-SNAP indeed directly interacts with furin (Fig. 2A), which was further confirmed by the fact that the furin-Flag was successfully pulled down by purified recombinant glutathione *S*-transferase-tagged α-SNAP protein (α-SNAP-GST) (Fig. 2B). Moreover, we found that α-SNAP-Δ231-250-Flag did not interact with furin-hemagglutinin (furin-HA) and α-SNAP-41-295-Flag interacted weakly with furin-HA (Fig. 2C).

A previous study identified a conserved membrane attachment site in the N-terminal domain of α-SNAP and found that mutation of two highly conserved phenylalanine residues (residues 27 and 28) disrupted the interaction between α-SNAP and membrane lipids (18). To test whether these two phenylalanine residues are important for the interaction between α-SNAP and furin, we changed them into the polar amino acid serine, as was done in the previous study (18), and designated the mutant α-SNAP-F27S/F28S-Flag. We then cotransfected α-SNAP-F27S/F28S-Flag and furin-HA into HEK293 cells. Coimmunoprecipitation assays showed that α-SNAP-F27S/F28S-Flag interacted weakly with furin-HA (Fig. 2D), similar to α-SNAP-41-295-Flag. α-SNAP-F27S/F28S-Flag had no effect on the cleavage of the SARS-CoV-2 S protein (Fig. 2E), indicating these two phenylalanine residues are necessary for the effect of α-SNAP on the cleavage of the SARS-CoV-2 S protein.

Furin has two domains that are indispensable for its protein-processing activity: a catalytic domain, which recognizes and cleaves the substrates, and the P domain, which is essential for pH and calcium modulation (19). To identify which domain(s) of furin interact(s) with α-SNAP, we generated two Flag-tagged truncated mutants of furin and tested their ability to bind α-SNAP-Myc in the coimmunoprecipitation assay (Fig. 2F). We found that deletion of the P domain abolished the interaction between furin and α-SNAP, whereas deletion of the catalytic domain of furin had no effect (Fig. 2G), indicating that α-SNAP interacts with the P domain of furin.

Newly assembled SARS-CoV-2 buds into the lumen of the endoplasmic reticulum and traffics to the *trans*-Golgi network to egress (20). The endoplasmic reticulum and the Golgi compartments are also sites of furin autoactivation (19). To track whether α-SNAP and furin interact in the endoplasmic reticulum and Golgi compartment, we performed a bimolecular fluorescence complementation (BiFC) assay in HEK293 cells (21). Briefly, the C-terminal end of furin was fused in-frame with the N-terminal 2 to 173 amino acids of the green fluorescent protein Venus (furin-VN), and the C-terminal of α-SNAP was fused with the C-terminal 154 to 238 amino acids of Venus (α-SNAP-VC). If the furin-VN and α-SNAP-VC interact after transfection into cells, the green BiFC signal will be detected. As shown in Fig. 2H and I, the BiFC signal colocalized with the endoplasmic reticulum marker calnexin and the Golgi marker GOLGA5 (Fig. 2H and I), indicating that furin and α-SNAP interact in endoplasmic reticulum and Golgi (Fig. 2H and I).

### α-SNAP regulates the catalytic activity of furin.

**FIG 3:**
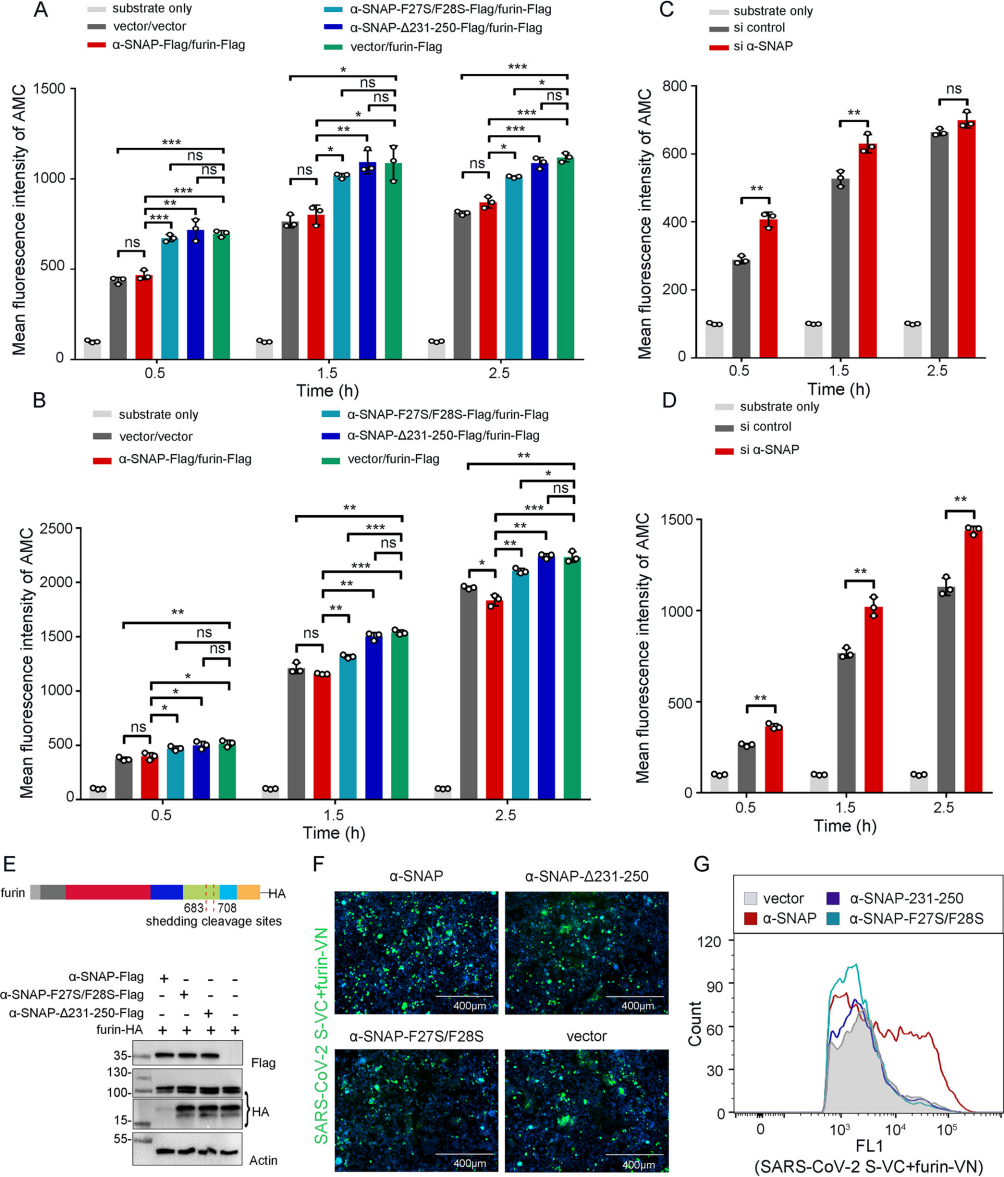
α-SNAP is a regulator of the protease activity of furin and inhibits SARS-CoV-2 infection in cells. (A and B) α-SNAP-Flag, α-SNAP-F27S/F28S-Flag, or α-SNAP-Δ231-250-Flag was cotransfected with furin-Flag into HEK293 cells for 48 h, and then the protease activity of furin in the cell culture supernatant (A) or cell lysate (B) was determined at the indicated times after adding Pyr-Arg-Thr-Lys-Arg-AMC substrate. (C and D) Furin activity in the cell culture supernatant (C) or cell lysate (D) was determined at the indicated time after adding Pyr-Arg-Thr-Lys-Arg-AMC substrate in α-SNAP-silenced HEK293 cells. (E) α-SNAP-Flag, α-SNAP-F27S/F28S-Flag, or α-SNAP-Δ231-250-Flag was cotransfected with furin-HA into HEK293 cells, and then furin was detected by immunoblotting for HA-tag. (F and G) SARS-CoV-2 S-VC and furin-VN were cotransfected with α-SNAP-Flag, α-SNAP-F27S/F28S-Flag, α-SNAP-Δ231-250-Flag, or vector into HEK293 cells. At 48 h posttransfection, the representative images were observed (F), and the fluorescence signal was detected by using flow cytometry (G). *n* = 3, means ± the SD. ns, not significant; *, *P < *0.05; **, *P < *0.01; ***, *P < *0.001 (Student test).

To investigate whether α-SNAP inhibits the catalytic activity of furin, we used a reporter substrate harboring the furin target motif Arg-Thr-Lys-Arg and monitored the release of fluorescent of 7-amino-4-methylcoumarin (AMC), as reported previously (22). HEK293 cells were transfected with empty vectors (vector/vector), α-SNAP-Flag/furin-Flag, α-SNAP-F27S/F28S-Flag/furin-Flag, α-SNAP-Δ231-250-Flag/furin-Flag, or vector/furin-Flag. The cell supernatants and cell lysates were collected at 48 h after transfection and mixed with the reporting substrate, and the fluorescence intensity of AMC was measured at different time points. The fluorescence intensity of AMC in the supernatant and cell lysate of cells transfected with α-SNAP-Δ231-250-Flag/furin-Flag and vector/furin-Flag was comparable but significantly higher than that of cells transfected with α-SNAP-Flag/furin-Flag or transfected with vector/vector (Fig. 3A and B), indicating that overexpression of furin could increase the cleavage of the reporting substrate and that α-SNAP, but not α-SNAP-Δ231-250, inhibits exogenous furin activity. Of note, overexpression of α-SNAP-F27S/F28S slightly inhibited the exogenous furin activity, which is consistent with the weak interaction between α-SNAP-F27S/F28S and furin, as shown in Fig. 2D.

We then performed an α-SNAP RNA interference (RNAi) assay in HEK293 cells to investigate the change in furin activity. The cell supernatants and cell lysates were collected at 60 h after transfection and mixed with the reporting substrate, and the fluorescence intensity of AMC was measured at different time points. The fluorescence intensity of AMC in the supernatant and cell lysate of α-SNAP-silenced cells was significantly higher than that of cells transfected with the si-control by 0.5, 1.5, and 2.5 h after the reaction occurred, although the fluorescence intensity of AMC in the supernatant of α-SNAP-silenced cells was comparable to that in the supernatant of cells transfected with the si-control at 2.5 h after the reaction occurred (Fig. 3C and D), indicating that knockdown of α-SNAP could increase the cleavage of the reporting substrate and that α-SNAP inhibits endogenous furin activity.

Coronavirus S protein is mainly cleaved by intracellular furin in the *trans*-Golgi network, and the coronavirus particles in the extracellular space might also be processed by plasma membrane-exposed or secreted furin (20, 23, 24). When furin is released into the extracellular space, its N-terminal containing catalytically active domain must be separated from the C-terminal transmembrane domain (from residues 683 or 708) via a cleavage step, which results in the generation of C-terminal furin fragments (ca. 15 to 20 kDa) (25, 26). We therefore investigated whether α-SNAP affects the separation of the N- and C-terminal fragments of furin. Immunoblotting analysis showed that the amount of the C-terminal furin fragment in cells transfected with α-SNAP-Flag/furin-HA was significantly lower than that in cells transfected with furin-HA, in cells transfected with α-SNAP-F27S/F28S-Flag/furin-HA, or in cells transfected with α-SNAP-Δ231-250-Flag/furin-HA (Fig. 3E). These results are consistent with the observation presented above that coexpression of α-SNAP and furin reduces the furin activity in cell supernatant, indicating that the interaction between α-SNAP and furin affects the release of furin into the extracellular space.

We further investigated whether α-SNAP could inhibit furin binding substrates. We performed BiFC assays to track the interaction between furin and the SARS-CoV-2 S protein in HEK293 cells. Green fluorescent signals were widely detected when furin-VN and SARS-CoV-2 S-VC were coexpressed in HEK293 cells, indicating that the SARS-CoV-2 S protein interacts with furin. When furin-VN and SARS-CoV-2 S-VC were coexpressed with α-SNAP in HEK293 cells, the green fluorescent signals were even stronger than those in cells expressing furin-VN and SARS-CoV-2 S-VC. In contrast, coexpression of α-SNAP-F27S/F28S or α-SNAP-Δ231-250 did not significantly affect the strength of the fluorescent signals in cells expressing furin-VN and SARS-CoV-2 S-VC (Fig. 3F), and these observations were confirmed by use of flow cytometry (Fig. 3G). Together, these results demonstrate that an increase in the level of α-SNAP reduces the catalytic activity of furin but does not affect the binding efficacy of furin for the SARS-CoV-2 S protein.

### Overexpression of α-SNAP inhibits SARS-CoV-2 infection in cells.

**FIG 4:**
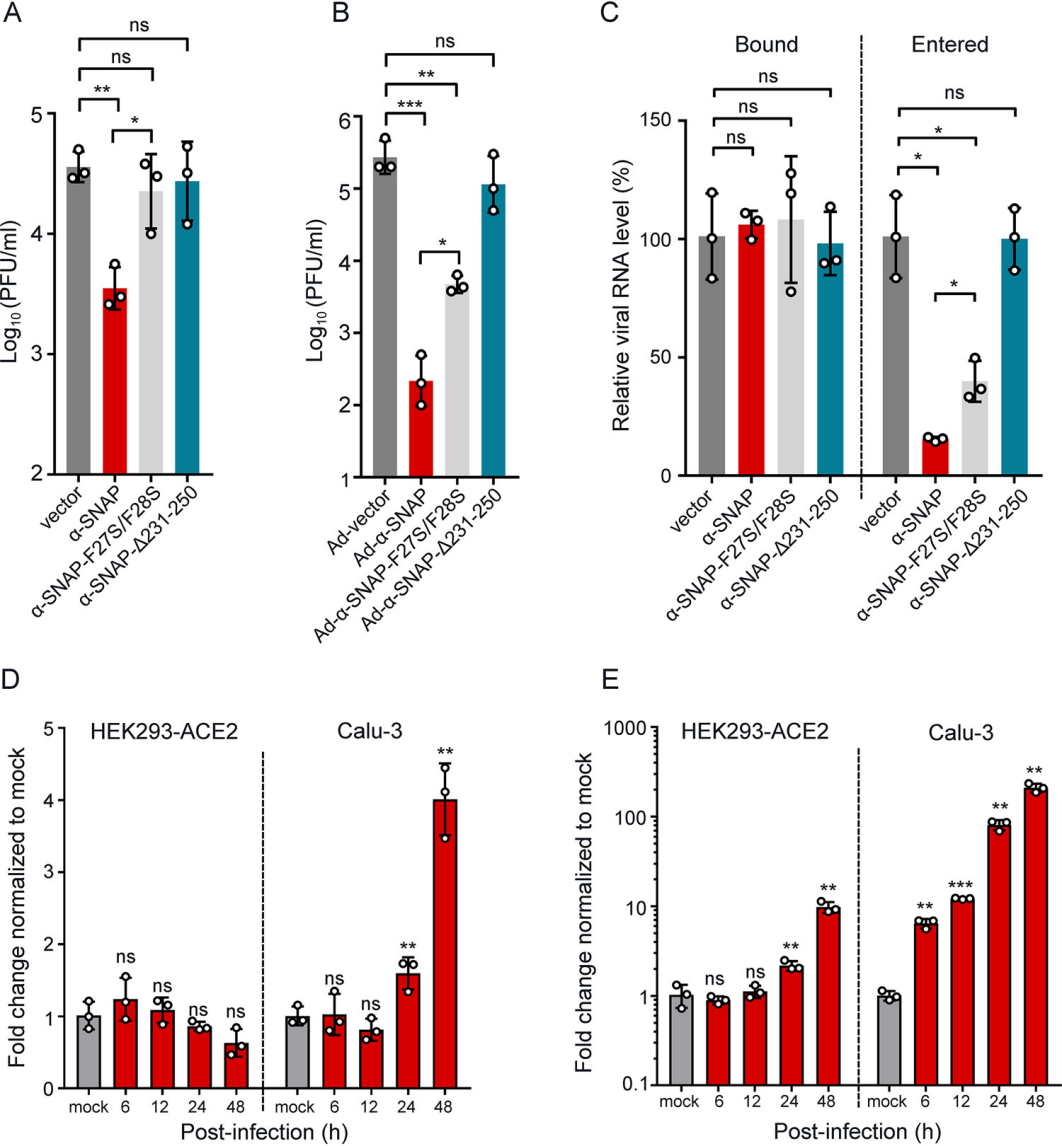
α-SNAP inhibits SARS-CoV-2 infection and is upregulated by SARS-CoV-2 stimulation in cells. (A) α-SNAP-Flag, α-SNAP-F27S/F28S-Flag, or α-SNAP-Δ231-250-Flag was transfected into HEK293-ACE2 cells. At 48 h posttransfection, the cells were infected with HRB25 (MOI = 0.01). The viral titers in the culture supernatant were detected by use of plaque assays at 24 h postinfection. (B) Calu-3 cells were transduced with Ad-vector, Ad-α-SNAP, Ad-α-SNAP-F27S/F28S, or Ad-α-SNAP-Δ231-250 at an MOI of 100 for 12 h and incubated for additional 12 h and then infected with HRB25 (MOI = 0.01). The cell culture supernatants were collected at 24 h postinfection to determine the viral titers by plaque assays. (C) α-SNAP-Flag-, α-SNAP-F27S/F28S-Flag-, or α-SNAP-Δ231-250-Flag-transfected HEK293-ACE2 cells were infected with HRB25 (MOI = 0.01). The cell culture supernatants were collected and titrated at 48 h postinfection and then used for evaluating their binding and entry to infect Calu-3 cells (MOI = 0.1). (D and E) HEK293-ACE2 cells or Calu-3 cells were infected with HRB25 (MOI = 0.5). At the indicated time of postinfection, the mRNA level of α-SNAP (D) or ISG56 (E) in the cell lysate were detected by qPCR. *n* = 3, means ± the SD. ns, not significant; *, *P < *0.05; **, *P < *0.01; ***, *P < *0.001 (Student test).

Efficient cleavage of the S protein is important for SARS-CoV-2 infection. To test whether overexpression of α-SNAP inhibits SARS-CoV-2 infection, α-SNAP-Flag-, α-SNAP-F27S/F28S-Flag-, or α-SNAP-Δ231-250-Flag-transfected HEK293 cells stably expressing human ACE2 (HEK293-ACE2) were infected with HRB25 (MOI = 0.01), and the supernatants of the infected cells were harvested at 24 h postinfection for viral titration. The viral titer in the α-SNAP-overexpressing cells was significantly lower than that in the vector-transfected control cells, whereas the viral titers in α-SNAP-F27S/F28S-Flag- and α-SNAP-Δ231-250-Flag-transfected cells were comparable to that in the vector-transfected control cells (Fig. 4A). We also performed overexpression and infection assay in Calu-3 cells, a human lung-derived cell line that is susceptible to SARS-CoV-2 infection. As transfection reagent or recombinant lentivirus is hard to transfect cDNA into Calu-3 cells, we altered to recombinant human adenovirus 5 (Ad) as the delivery vector. Calu-3 cells were transduced with Ad-α-SNAP, Ad-α-SNAP-F27S/F28S, or Ad-α-SNAP-Δ231-250 and then infected with HRB25 (MOI = 0.01). The supernatants of the infected cells were harvested at 24 h postinfection for viral titration. The viral titer in α-SNAP-overexpressing cells was significantly lower than that in the vector-transduced control cells, whereas there was no significant difference compared to that in α-SNAP-Δ231-250-Flag-overexpressing cells (Fig. 4B). Of note, the viral titer in α-SNAP-F27S/F28S-overexpressing cells was significantly lower than that in control cells, whereas higher than that in α-SNAP-overexpressing cells. These results demonstrate that overexpression of α-SNAP inhibits the cleavage of the SARS-CoV-2 S protein and thereby inhibits SARS-CoV-2 replication.

The cleavage of S protein by furin contributes to SARS-CoV-2 entry (17, 27, 28). We next investigated whether the entry of SARS-CoV-2 produced in α-SNAP-overexpressing cells and control cells is different. HEK293-ACE2 cells transfected with α-SNAP-Flag, α-SNAP-F27S/F28S-Flag, α-SNAP-Δ231-250-Flag, or vector were infected with HRB25 (MOI = 0.01). The supernatants were harvested at 48 h postinfection and titrated in Vero-E6 cells. The titrated viruses in the supernatants were then used for evaluating their binding and entry in Calu-3 cells. Briefly, Calu-3 cells were incubated with viruses from the supernatants described above (MOI = 0.1) at 4°C for 1 h and then at 37°C for 6 h. After incubation at 4°C for 1 h or at 37°C for 6 h, the level of viral RNA in cell lysates was detected by qPCR to reflect the number of bound virus or entered virus. We found that the RNA level of the bound viruses in cells received different inoculums was comparable; however, the RNA level of the entered viruses in cells received the inoculum from α-SNAP-Flag- and α-SNAP-F27S/F28S-Flag-transfected cells was significantly lower than that from α-SNAP-Δ231-250-Flag- or vector-transfected cells (Fig. 4C). These results indicate that overexpression of α-SNAP reduces SARS-CoV-2 infection mainly through inhibiting furin-mediated cleavage of S protein and thereby decreasing entry of progeny virus.

### α-SNAP is upregulated by interferon stimulation.

The above studies demonstrated that overexpression of α-SNAP inhibits SARS-CoV-2 replication in cells. We then sought to determine whether α-SNAP upregulated in SARS-CoV-2-infected cells to perform an antiviral function. To answer this question, we evaluated the mRNA level of α-SNAP in the SARS-CoV-2-infected HEK293-ACE2 cells or Calu-3 cells. The α-SNAP mRNA level is significantly increased upon SARS-CoV-2 infection in Calu-3 cells, whereas it is not significantly increased upon SARS-CoV-2 infection in HEK293-ACE2 cells (Fig. 4D).

**FIG 5:**
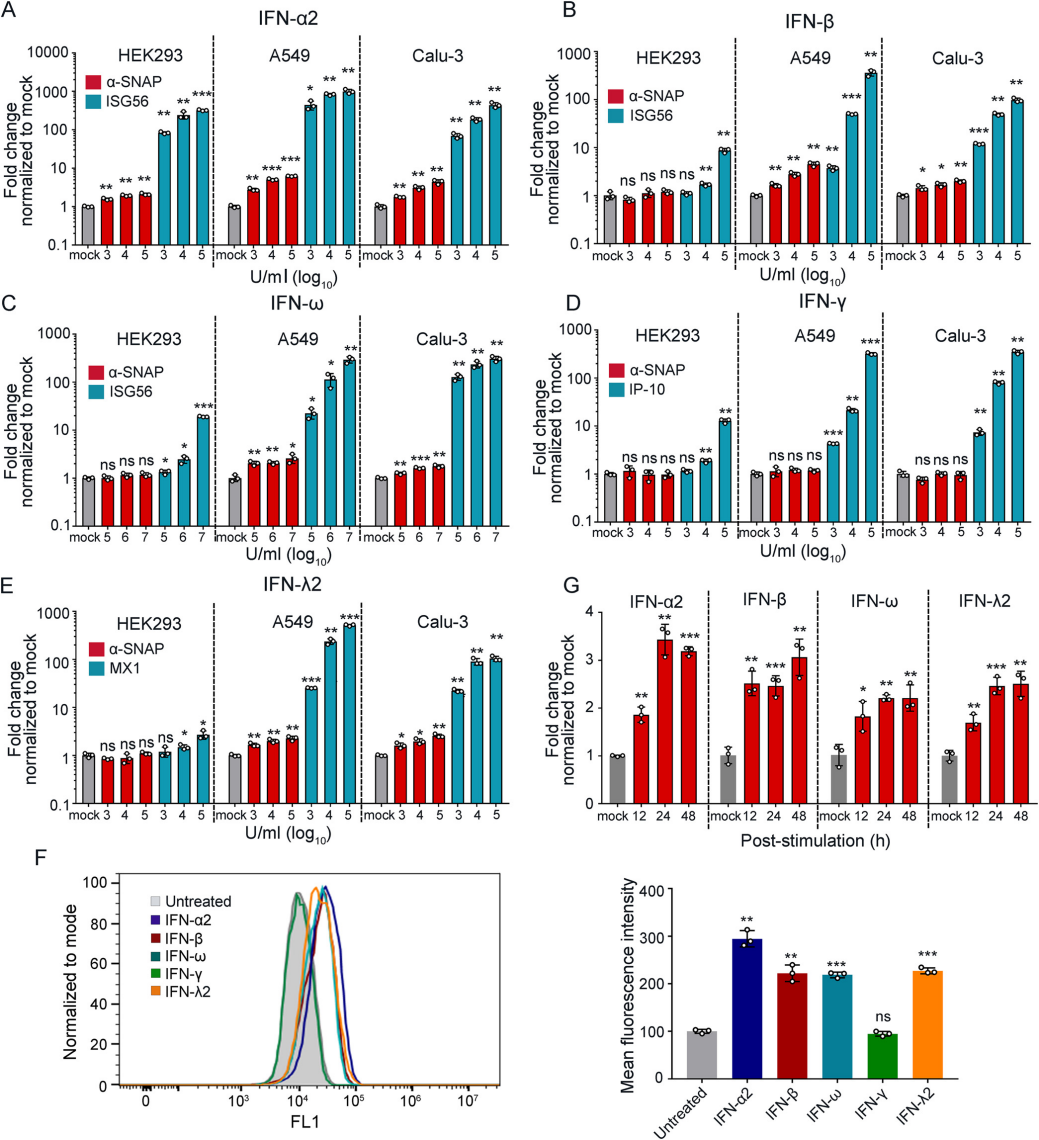
α-SNAP is upregulated by type I and type III IFNs. (A to E) HEK293 cells, A549 cells, or Calu-3 cells were stimulated with different concentrations of IFN-α2 (A), IFN-β (B), IFN-ω (C), IFN-γ (D), or IFN-λ2 (E) for 24 h, and then the cell lysates were collected to detect α-SNAP mRNA and ISG56 mRNA, IP-10 mRNA, or MX1 mRNA relative to human 28s rRNA by qPCR, respectively. (F) A549 cells were stimulated with IFN-α2 (10^4^ U/mL), IFN-β (10^4^ U/mL), IFN-ω (10^6^ U/mL), IFN-γ (10^4^ U/mL), or IFN-λ2 (10^4^ U/mL) for 24 h, and then the expression of α-SNAP was detected by using flow cytometry. (G) A549 cells were stimulated with IFN-α2 (10^4^ U/mL), IFN-β (10^4^ U/mL), IFN-ω (10^6^ U/mL), or IFN-λ2 (10^4^ U/mL). At the indicated time poststimulation, α-SNAP mRNA relative to human 28s rRNA in the cell lysates was measured by qPCR. *n* = 3, means ± the SD. ns, not significant; *, *P < *0.05; **, *P < *0.01; ***, *P < *0.001 (Student test).

Type I interferon (IFN) plays an important role in the host antiviral response, and different cellular factors are upregulated by IFN stimulation to then exert antiviral effects. After infection of SARS-CoV-2, the interferon-stimulated gene (ISG) 56 RNA level in Calu-3 cells is significantly higher than that in HEK293-ACE2 cells (Fig. 4E), which indicates that SARS-CoV-2 infection triggering the expression of IFN in Calu-3 cells is stronger than that in HEK293-ACE2 cells. We therefore tested whether upregulation of α-SNAP in SARS-CoV-2-infected cells was stimulated by IFN. If IFN stimulates the upregulation of α-SNAP, this would partially explain why SARS-CoV-2 is more sensitive to type I IFN than SARS-CoV (29) because SARS-CoV has no furin cleavage motif in its S protein and upregulated α-SNAP has no antiviral effect in SARS-CoV infection. We therefore stimulated different cells with different types of IFNs and tested the α-SNAP mRNA level by qPCR. Three well-known ISGs—ISG56, IP-10, and MX1—that are respectively induced by the stimulation of type I, type II, and type III IFNs were selected as positive controls. We found that type I (IFN-α2, IFN-β, and IFN-ω) and type III (IFN-λ2) IFNs, but not type II IFN (IFN-γ), can upregulate the expression of α-SNAP in HEK293, A549, and Calu-3 cells (Fig. 5A to E); these results were confirmed by flow cytometry (Fig. 5F). We further found that the expression of α-SNAP in type I IFN- or type III IFN-treated A549 cells progressively increased over the stimulation time (Fig. 5G). These results demonstrate that type I and type III IFNs can upregulate the expression of α-SNAP in cells.

## DISCUSSION

α-SNAP is a well-known component of the vesicle trafficking machinery and is required for the final step of all intracellular membrane trafficking events (15). In the present study, we found that α-SNAP is upregulated in SARS-CoV-2-infected cells and exerts antiviral activity by inhibiting the cleavage of the SARS-CoV-2 S protein and restricting SARS-CoV-2 infection in cells. We further revealed that α-SNAP is an important regulator of furin activity and inhibits the catalytic activity of furin by directly interacting with its P domain. Moreover, the cellular α-SNAP level could be upregulated by stimulation of type I and type III IFNs. Our study thus identifies a novel function of α-SNAP as an ISG that plays an antiviral role during SARS-CoV-2 infection.

Numerous ISGs have been identified in the past decades (30). Some ISGs are expressed basally in the absence of IFN stimulation, whereas others appear to be expressed only during an IFN response (30). A previous study indicated that plasminogen activator inhibitor, which inhibits influenza A virus glycoprotein cleavage by targeting extracellular airway proteases, is constitutively expressed in cells, and could be further upregulated by IFN and other cytokines (31). α-SNAP is expressed at a certain level in normal cells to play an important role in intracellular membrane trafficking events. Our study shows that α-SNAP is upregulated in SARS-CoV-2-infected cells and can be upregulated by both type I and type III IFNs. It remains to be investigated whether α-SNAP can also be upregulated by other cytokines.

Several viral pathogens require furin for the cleavage and maturation of their envelope glycoproteins during infection. The glycoproteins of Zaire Ebola virus, Marburg virus, and MERS-CoV all carry a cleavage motif that is recognized by furin (8, 23). In this study, we demonstrated that α-SNAP also regulate the cleavage of the glycoproteins of these viruses (see Fig. S3). Therefore, it is reasonable to speculate that α-SNAP may also play an antiviral role during infections caused by other viral pathogens that carry the furin motif in their glycoproteins.

10.1128/mBio.02443-21.3 FIG S3 Overexpression of α-SNAP inhibits cleavage of ZEBOV GP, MARV GP, and the MERS-CoV S protein. (A to C) α-SNAP-Flag was cotransfected with ZEBOV GP-Myc, MARV GP-Myc or MERS-CoV S-Myc into HEK293 cells, and then ZEBOV GP (A), MARV GP (B) or the MERS-CoV S protein (C) was detected by immunoblotting for Myc tag. (D to F) α-SNAP-Flag or α-SNAP-F27S/F28S-Flag was cotransfected with ZEBOV GP-Myc, MARV GP-Myc, or MERS-CoV S-Myc into HEK293 cells, and then ZEBOV GP (D), MARV GP (E), or the MERS-CoV S protein (F) was detected by immunoblotting for Myc-tag. (G to I) α-SNAP-Flag or α-SNAP-Δ231-250-Flag was cotransfected with ZEBOV GP-Myc, MARV GP-Myc, or MERS-CoV S-Myc into HEK293 cells, and then ZEBOV GP (G), MARV GP (H), or the MERS-CoV S protein (I) was detected by immunoblotting for Myc tag. Download FIG S3, TIF file, 2.9 MB. Copyright © 2022 Wang et al. 2022 Wang et al. https://creativecommons.org/licenses/by/4.0/ This content is distributed under the terms of the Creative Commons Attribution 4.0 International license.

In summary, our study has demonstrated that α-SNAP is an IFN-stimulated gene that exerts antiviral activity by inhibiting furin activity. These findings open a new door for the development of antiviral strategies against SARS-CoV-2 and other furin-dependent pathogens.

## MATERIALS AND METHODS

### Cell lines.

All cells were cultured at 37°C with 5% CO_2_ in a humidified incubator. HEK293 cells (ATCC CRL-1573), HEK293E cells (ATCC, CRL-10852) and Vero-E6 cells (ATCC, CRL-1586) were maintained in Dulbecco modified Eagle medium (DMEM), A549 cells (ATCC, CCL-185) were maintained in F-12K Nutrient Mixture, and Calu-3 cells (ATCC, HTB-55) were grown in Eagle minimal essential medium (MEM). The stable HEK293 cell line expressing human ACE2 (HEK293-ACE2) was generated by transducing the cells with an ACE2-expressing lentiviral vector and selection with 4 μg/mL puromycin. The selected cells were subsequently maintained in DMEM with 2 μg/mL puromycin. Media were supplemented with 10% fetal bovine serum (FBS), 1% penicillin/streptomycin (P/S), and l-glutamine.

### Viruses.

SARS-CoV-2/HRB25/human/2020/CHN (HRB25, GISAID accession no. EPI_ISL_467430) was maintained in our laboratory. All experiments with infectious SARS-CoV-2 were performed in the biosafety level 4 facilities at the Harbin Veterinary Research Institute (HVRI) of the Chinese Academy of Agricultural Sciences (CAAS).

The recombinant nonreplicating E1/E3 deleted human adenovirus type 5 vectors (Ad-vector) expressing α-SNAP, α-SNAP-F27S/F28S, or α-SNAP-Δ231-250 were generated in HEK293E cells using a kit according to the instructions of the manufacturer (AdEasy adenoviral vector system). The resulting recombinants were named Ad-α-SNAP, Ad-α-SNAP-F27S/F28S, and Ad-α-SNAP-Δ231-250, respectively. The viral stocks were propagated in HEK293E cells and purified by ultracentrifugation (20,000 rpm for 1.5 h). The viral titers were determined as the 50% tissue culture infectious dose.

### Plasmids.

The genes for SARS-CoV-2 S (GenBank MN908947.3) and its S1 subunit (amino acids [aa] 14 to 685), SARS-CoV S (GenBank AAP13441.1), MERS-CoV S (GenBank KF186567.1), Zaire Ebola virus (ZEBOV) glycoprotein (GP; GenBank KM655246.1), and Marburg virus (MARV) GP (GenBank NC001608.3) were subcloned into the pCAGGS-Myc expression vector in-frame. The genes for α-SNAP (GenBank NM_003827.4), β-SNAP (GenBank NM_001283018.2), γ-SNAP (GenBank NM_003826.3), and NSF (GenBank NM_006178.4) were synthesized by Comate Bioscience Co., Ltd. The furin gene was generated by PCR using human cDNA as a template. α-SNAP and furin were subcloned into the pCAGGS-Myc or pCAGGS-HA expression vector in-frame, respectively. α-SNAP and a series of its mutants, β-SNAP, γ-SNAP, and furin and a series of its mutants were subcloned into the pCAGGS-Flag expression vector in-frame. Furin was inserted into the pcDNA3.1-VN vector in-frame. SARS-CoV-2 S and α-SNAP were inserted into the pcDNA3.1-Flag-VC vectors in-frame. The integrity of all constructs was confirmed by sequencing.

### Cell membrane protein isolation.

HEK293 cells were harvested and membrane proteins were isolated by using a Minute plasma membrane protein isolation and cell fractionation kit (Invent Biotechnologies). The pellet was collected for further studies.

### Western blotting.

At 48 h posttransfection, cells were lysed with 1% NP-40 phosphate-buffered saline (PBS; Beyotime Biotechnology) for 1 h and cleared by centrifugation at 12,000 rpm for 20 min at 4°C. Clarified cell lysate was diluted in denaturing sodium dodecyl sulfate (SDS) gel loading buffer and boiled for 15 min. After denaturing, the samples were separated on a 4 to 12% SDS-PAGE gel (GenScript) and blotted onto polyvinylidene difluoride membrane (Merck-Millipore). The membrane was blocked with 5% skim milk in PBS containing 0.1% Tween 20, followed by incubation with the following primary antibodies: rabbit anti-HA MAb (1:500; Abcam), mouse anti-Flag MAb (1:1,000; GenScript), rabbit anti-Myc pAb (1:1,000; GenScript), rabbit anti-GST pAb (1:1,000; GenScript), or mouse anti-β-actin MAb (1:1,000; Zsbio). After washing, the membrane was incubated with horseradish peroxidase-conjugated goat anti-mouse pAb antibody (1:5,000; GenScript) or goat anti-rabbit pAb antibody (1:5,000; GenScript) and detected by using enhanced chemiluminescence (ECL) reagent (Merck Millipore, WBLUR0500).

To investigate inhibitory effects on the cleavage of different viral glycoproteins, α-SNAP-Flag, a series of α-SNAP mutants, β-SNAP-Flag or γ-SNAP-Flag and SARS-CoV-2 S-Myc, SARS-CoV S-Myc, MERS-CoV S-Myc, ZEBOV GP-Myc or MARV GP-Myc were cotransfected into HEK293 cells. At 48 h posttransfection, the cells were lysed and subjected to Western blotting.

### Coimmunoprecipitation assay.

Cells were transfected by using ExFect transfection reagent (Vazyme) according to the manufacturer’s instructions. α-SNAP-Flag and SARS-CoV-2 S-Myc were cotransfected into HEK293 cells. α-SNAP-Flag or a series of mutations of α-SNAP-Flag and furin-HA were cotransfected into HEK293 cells, respectively. Furin-Flag or a series of mutations of furin-Flag and α-SNAP-Myc were cotransfected into HEK293 cells, respectively. At 48 h posttransfection, cells were lysed with 1% NP-40 PBS buffer for 1 h at 4°C. The supernatant was collected and then mixed with protein G-agarose (Roche) for 4 h at 4°C. After centrifugation, the supernatant was collected and incubated overnight with anti-Flag antibody-conjugated agarose beads (Sigma) for 6 h at 4°C. After five washes with prechilled 1% NP-40 PBS buffer, the beads were resuspended and subjected to SDS-PAGE.

For mass spectrometry identification of SARS-CoV-2 S1-Flag interacting proteins, four 15-cm dishes of HEK293 cells were transfected with pCAGGS encoding Flag-tagged protein for 48 h. Cell membrane proteins were extracted as described above and then subjected to coimmunoprecipitation assays. The samples were separated on 4 to 12% SDS-PAGE gels (GenScript) and subsequently analyzed by using mass spectrometry. Three independent biological replicates were performed for each bait protein. Affinity purification coupled to mass spectrometry analysis was performed and analyzed by using Jingjie PTM BioLab.

### Pulldown assay.

The purified GST-tagged α-SNAP (FriendBio Technology) was mixed with 20 μL of glutathione-Sepharose 4B beads (GE Healthcare Bioscience) for 4 h. After a washing step, the beads were mixed with lysis supernatant from furin-Flag-transfected HEK293 cells for 6 h at 4°C. The beads were then washed five times with prechilled 1% NP-40 PBS buffer. The beads were resuspended and subjected to SDS-PAGE.

### Confocal microscopy.

HEK293 cells were seeded on a glass bottom cell culture dish (Nest Biotechnology) 12 h before transfection. Cells were cotransfected with the α-SNAP-VC/furin-VN pair. At 24 h posttransfection, the cells were fixed with 4% paraformaldehyde. The cells were then permeabilized with 0.1% Triton X-100 and blocked with 1% bovine serum albumin (BSA). The cells were incubated with rabbit anti-GOLGA5 (1:500; Sigma) or rabbit anti-ER marker (1:500; Abcam) primary antibody for 1 h at room temperature. After three washes with PBS, Alexa Fluor 647-conjugated secondary antibody (1:500; Invitrogen) was added, followed by incubation for 1 h at room temperature. After another three washes with PBS, the cells were stained with DAPI (1:2,000; Sigma) for 10 min and washed five times with PBS at room temperature before being observed under a confocal microscope (Zeiss LSM880).

### Furin activity assay.

HEK293 cells were seeded in 24-well plates 12 h before transfection. Furin-Flag was cotransfected with α-SNAP-Flag, α-SNAP-F27S/F28S-Flag, α-SNAP-Δ231-250-Flag, or vector into HEK293 cells. At 48 h posttransfection, cell culture supernatants were harvested. The cells were washed with PBS, 500 μL of H_2_O per well was added, and the cells were then lysed by several freeze-thaw cycles. After 15 min of incubation on ice, cell lysates were clarified by centrifugation. Portions (20 μL) of cell culture supernatants were incubated with Pyr-Arg-Thr-Lys-Arg-7-amido-4-methylcoumarin (AMC) substrate (1 nmol; Bachem) in 80 μL of reaction buffer (100 mM HEPES [pH 7.5], 0.5% Triton X-100, 1 mM CaCl_2_, 1 mM 2-mercaptoethanol). Assays were performed at 37°C, and the reactions were terminated at the indicated time by dilution with 100 μL of 25 mM ZnCl_2_. The mean fluorescence intensity of furin-cleaved AMC substrate was determined by using the Enspire Multiscan Spectrum (355-nm excitation and 460-nm emission; Perkin-Elmer).siRNA transfections were performed in 24-well plates using Lipofectamine RNAiMAX transfection reagent (Thermo Fisher Scientific) according to the manufacturer’s instructions. Briefly, siRNA (sense, 5′-GCUCCUUCCCUAAUGCUUUTT-3′; antisense, 5′-AAAGCAUUAGGGAAGGAGCAG-3′; 5 μM, 3 μL/well; Thermo Fisher Scientific) targeting α-SNAP or nontargeting siRNA (Thermo Fisher Scientific) was mixed with 117 μL of Opti-MEM medium (Invitrogen) containing 0.8 μL of Lipofectamine RNAiMAX transfection reagent on 24-well plates. After a 30-min incubation at room temperature, the mixture was added dropwise to the HEK293 cells. At 60 h posttransfection, cell culture supernatants and cell lysates were collected to detect the furin activity as described above.

### Flow cytometry.

For BiFC assays, α-SNAP-Flag, α-SNAP-231-250-Flag, α-SNAP-F27S/F28S-Flag, or vector was cotransfected with the furin-VN/SARS-CoV-2 S-VC pair into HEK293 cells. At 48 h posttransfection, representative images of each well were obtained using an inverted fluorescence microscope (Invitrogen). The cells were then thoroughly washed with PBS and trypsinized with 0.25% trypsin (without EDTA). The cells were collected, washed three times with fluorescence-activated cell sorting (FACS) wash buffer (PBS containing 3% FBS and 0.1% NaN_3_), and then fixed with 4% paraformaldehyde at room temperature for 10 min. The cells were permeabilized with 0.1% Triton X-100 in PBS for 15 min and then blocked with 1% BSA for 1 h. The cells were next incubated with mouse anti-Flag MAb (1:500; Sigma, SLCD6338) for 1 h. After three washes with FACS wash buffer, Alexa Fluor 647-conjugated goat anti-mouse IgG secondary antibody (1:500; Invitrogen, A21236) was added, followed by incubation for 1 h at room temperature. After three washes with FACS wash buffer, the mean fluorescence intensities (MFIs) of the samples were obtained using a FC500 flow cytometer (Beckman Coulter). We first calculated the Alexa Fluor 647 MFIs of samples and gated Alexa Fluor 647-positive cells, and then we calculated the green fluorescent protein MFI of samples in Alexa Fluor 647-positive cells. The data were analyzed using FlowJo software (FlowJo VX).

IFN-stimulated A549 cells were processed as described above, except that mouse anti-α/β-SNAP MAb (1:500; Santa Cruz, sc-48349) was used as the primary antibody and FITC-conjugated goat anti-mouse IgG (1:100; Zsbio, ZF-0312) served as the secondary antibody, and were then analyzed by flow cytometry.

### Plaque assay.

Serial dilutions of supernatants from infected cells were added to Vero-E6 cell monolayers and adsorbed for 1 h at 37°C. After a washing step, plaque medium was overlaid on the cells, followed by incubation at 37°C. After 48 h of incubation, the cell monolayers were stained with crystal violet, and the plaques were counted.

### Virus infection assay.

HEK293-ACE2 cells or Calu-3 cells seeded onto 24-well plates were infected with HRB25 (MOI = 0.01 for HEK293-ACE2 cells; MOI = 0.5 for Calu-3 cells) in a 100-μL volume for 1 h at 37°C. The cells were washed three times with DMEM (HEK293-ACE2 cells) or MEM (Calu-3 cells) containing 2% FBS; 500 μL of DMEM or MEM containing 2% FBS was then added to the cells for further studies.

To determine the mRNA level of α-SNAP or ISG56 in SARS-CoV-2-infected cells, Calu-3 cells or HEK293-ACE2 cells were infected by HRB25 at an MOI of 0.5. At 6, 12, 24, and 48 h postinfection, the cell lysates were respectively collected to determine the mRNA level of α-SNAP or ISG56 by qPCR.

### Overexpression and infection assays.

HEK293-ACE2 cells were seeded onto 24-well plates. The cells were transfected with 0.5 μg of empty vector, α-SNAP-Flag, α-SNAP-F27S/F28S-Flag, or α-SNAP-Δ231-250-Flag using ExFect transfection reagent (Vazyme) and according to the manufacturer’s instructions. At 48 h posttransfection, the cells were infected with HRB25 (MOI = 0.01) for 1 h at 37°C. Cell culture supernatants were harvested at 24 and 48 h postinfection, respectively, and the viral titers were determined by performing plaque assays. Calu-3 cells were incubated with the viruses from cell culture supernatants at 48 h postinfection (MOI = 0.1) for 1 h at 4°C and then shifted to 37°C. After incubation at 4°C for 1 h or at 37°C for 6 h, the RNA level of SARS-CoV-2 N gene in cell lysates was detected by qPCR to reflect the number of bound virus or entered virus.

Calu-3 cells were seeded onto 24-well plates and then immediately transduced with Ad-vector, Ad-α-SNAP, Ad-α-SNAP-F27S/F28S, or Ad-α-SNAP-Δ231-250 at an MOI of 100 for 12 h at 37°C. The cells were washed three times with MEM containing 10% FBS, and 500 μL of MEM containing 10% FBS was added to the cells, followed by incubation at 37°C for additional 24 h. At 24 h postinfection, the cells were infected with HRB25 (MOI = 0.01). At 24 h postinfection, cell culture supernatants were harvested to determine the viral titers by plaque assays.

### Real-time quantitative PCR.

Total RNA was isolated from cells by using TRIzol reagent (Thermo Fisher) and reverse transcribed into cDNA using HiScript II Q RT SuperMix for real-time quantitative PCR (qPCR; Vazyme) according to the manufacturer’s instructions. The transcript levels were determined by using SYBR green qPCR Master Mix (Vazyme) on a QuantStudio 5 fluorescent quantitative PCR instrument (Applied Biosystems). The 2^–ΔΔ^*^CT^* method was used to calculate the relative gene expression level, with 28S rRNA. The qPCR primers used were as follows: α-SNAP (human), forward (5′-ATTGACATCTACGAACAGGTGGG-3′) and reverse (5′-ATGCAGAAGTGG CAGAGGGC-3′); 28S rRNA (human), forward (5′-GGGTGGTAAACTCCATCTAAGG-3′) and reverse (5′-GCCCTCTTGAACTCTCTCTTC-3′); ISG-56 (human), forward (5′-AACACCCACTTCTGTCTTACTGC-3′) and reverse (5′-TTTCTGTGATTGCCTGCTTCTAT-3′); MX1 (human), forward (5′-CTGGTGCTGAAACTGAAGAAAC-3′) and reverse (5′-TACCTCTGAAGCATCCGAAATC-3′); and IP-10 (human), forward (5′-CCATTCTGATTTGCTGCCTTATC-3′) and reverse (5′-TACTAATGCTGATGCAGGTACAG-3′) and the SARS-CoV-2 N gene-specific primers (forward, 5′-GGGGAACTTCTCCTGCTAGAAT-3′; reverse, 5′-CAGACATTTTGCTCTCAAGCTG-3′).

### Interferon induction assay.

HEK293, A549, and Calu-3 cells were incubated with different concentrations of recombinant human IFN-α2 (10^3^, 10^4^, and 10^5^ U/mL; BioLegend), recombinant human IFN-β (10^3^, 10^4^, and 10^5^ U/mL; PeproTech), recombinant human IFN-ω (10^5^, 10^6^, and 10^7^ U/mL; PeproTech), recombinant human IFN-γ (10^3^, 10^4^, and 10^5^ U/mL; BioLegend), and recombinant human IFN-λ2 (10^3^, 10^4^, and 10^5^ U/mL; PeproTech), respectively. At 24 h poststimulation, the cells were harvested, and the gene expression levels of α-SNAP, ISG56, IP-10, and MX1 were determined by qPCR.

A549 cells were incubated with IFN-α2 (10^4^ U/mL), IFN-β (10^4^ U/mL), IFN-γ (10^4^ U/mL), IFN-λ2 (10^4^ U/mL), and IFN-ω (10^6^ U/mL), respectively. At 12, 24, and 48 h poststimulation, the cells were harvested to determine α-SNAP mRNA levels by qPCR. The α-SNAP protein expression levels at 24 h poststimulation were determined by using flow cytometry.

### Statistics and reproducibility.

All data are expressed as arithmetic means ± the standard deviations (SD). Independent biological replicates (*n* = 3) were used for all experiments unless otherwise indicated. *P* values were calculated using an unpaired two-tailed Student *t* test (values are indicated by asterisks in the figures: *, *P < *0.05; **, *P < *0.01; and ***, *P < *0.001). Calculations were performed using GraphPad Prism software.
