# Supplementary figures and images for: Alpha-Soluble NSF Attachment Protein Prevents the Cleavage of the SARS-CoV-2 Spike Protein by Functioning as an Interferon-Upregulated Furin Inhibitor
Source: mBio. 2022 Jan 11;13(1):e02443-21. doi: 10.1128/mbio.02443-21 (PMC8749436; doi:10.1128/mbio.02443-21)

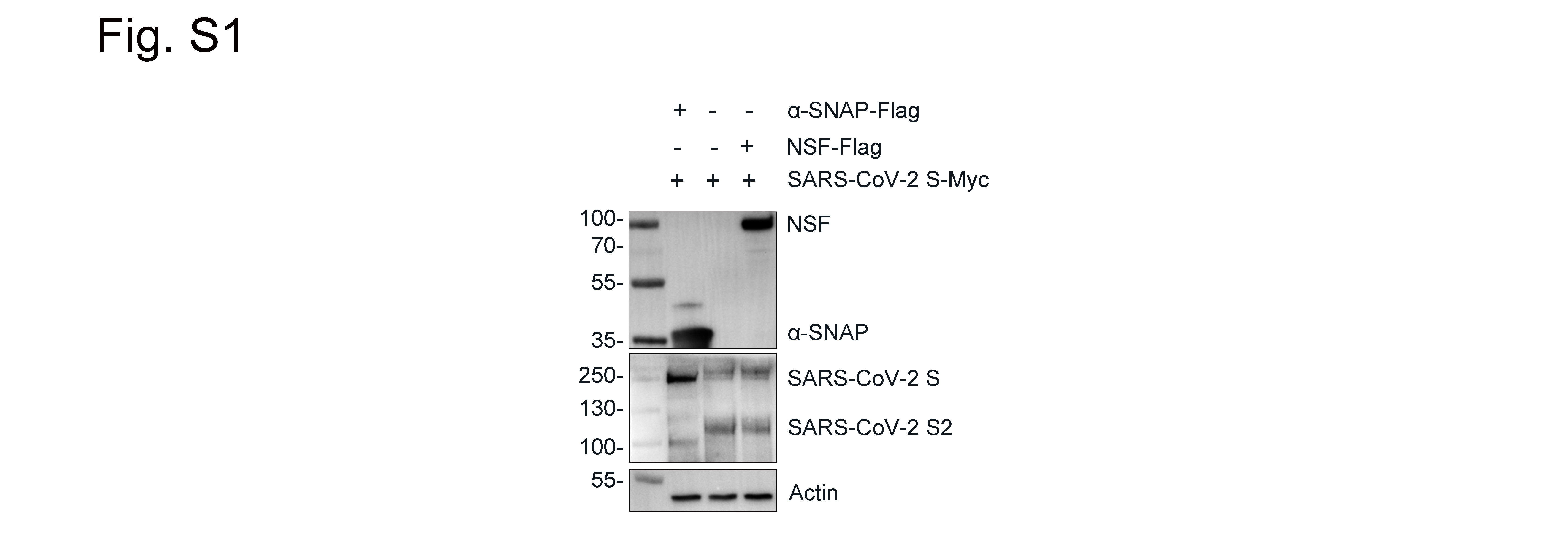

Supplement: FIG S1 [file mbio.02443-21-sf001.tif]

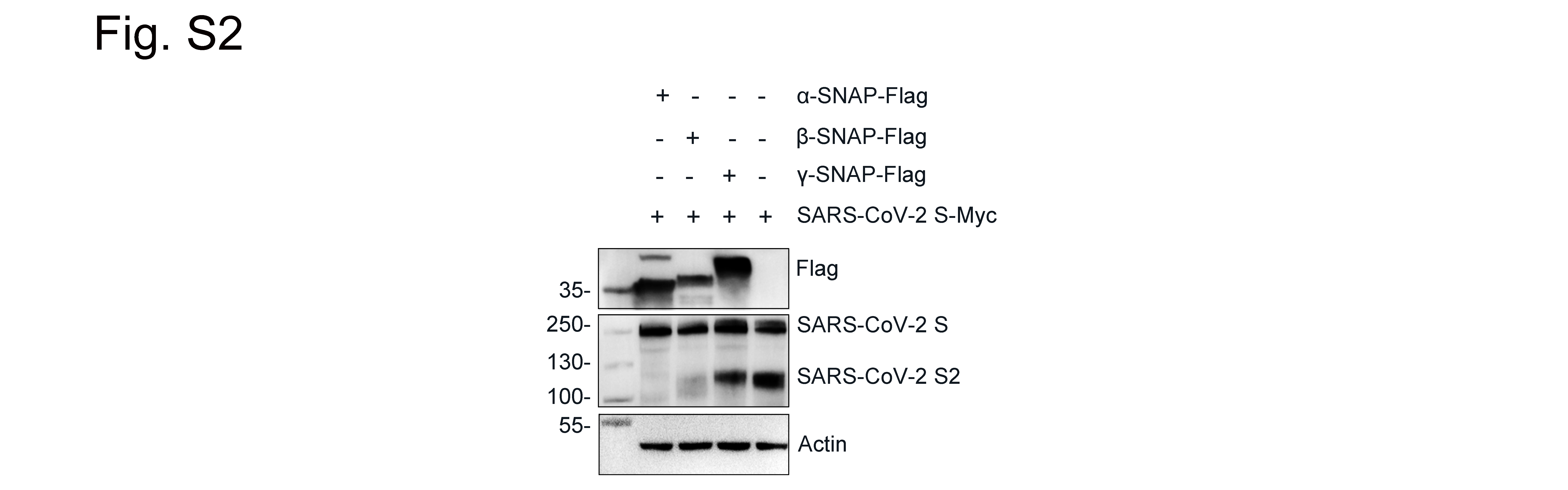

Supplement: FIG S2 [file mbio.02443-21-sf002.tif]

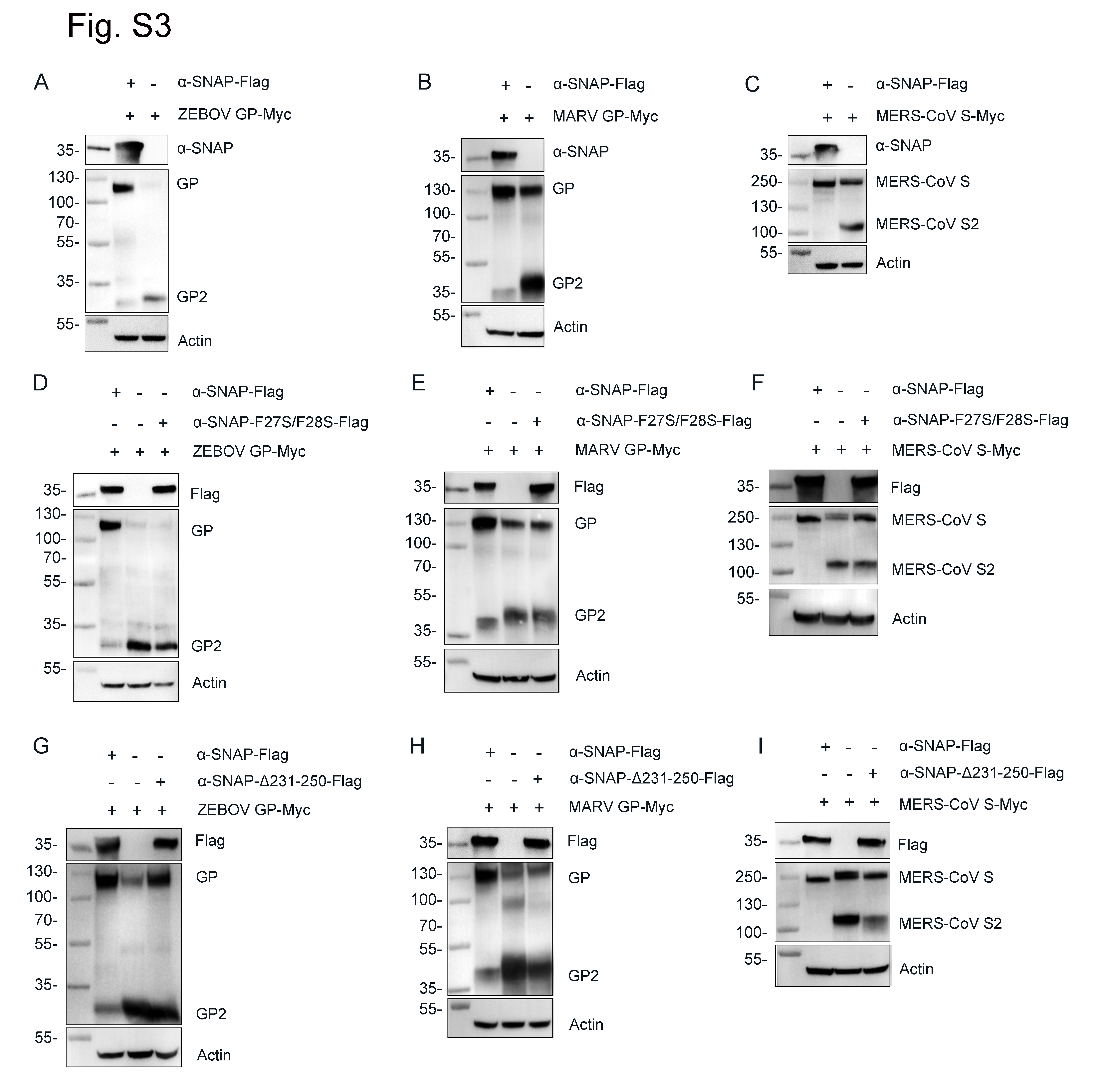

Supplement: FIG S3 [file mbio.02443-21-sf003.tif]
